# Preferences for care towards the end of life when decision-making capacity may be impaired: A large scale cross-sectional survey of public attitudes in Great Britain and the United States

**DOI:** 10.1371/journal.pone.0172104

**Published:** 2017-04-05

**Authors:** Gemma Clarke, Elizabeth Fistein, Anthony Holland, Matthew Barclay, Pia Theimann, Stephen Barclay

**Affiliations:** 1 Cambridge Institute of Public Health, Department of Public Health and Primary Care, University of Cambridge, Cambridge Biomedical Campus, Forvie Site, Robinson Way, Cambridge, United Kingdom; 2 Cambridge Intellectual and Developmental Disabilities Research Group, Department of Psychiatry, University of Cambridge, Douglas House, Cambridge, United Kingdom; Taipei Veterans General Hospital, TAIWAN

## Abstract

**Background:**

There is continuing public debate about treatment preferences at the end of life, and the acceptability and legal status of treatments that sustain or end life. However, most surveys use binary yes/no measures, and little is known about preferences in neurological disease when decision-making capacity is lost, as most studies focus on cancer. This study investigates changes in public preferences for care towards the end of life, with a focus on measures to sustain or end life.

**Methods:**

Large-scale international public opinion surveys using a six-stage patient vignette, respondents chose a level of intervention for each stage as health and decision-making capacity deteriorated. Cross-sectional representative samples of the general public in Great Britain and the USA (N = 2016). Primary outcome measure: changes in respondents’ preferences for care, measured on a four-point scale designed before data collection. The scale ranged from: maintaining life at all costs; to intervention with agreement; to no intervention; to measures for ending life.

**Results:**

There were no significant differences between GB and USA. Preference for measures to sustain life at all costs peaked at short-term memory loss (30.2%, n = 610). Respondents selecting ‘measures to help me die peacefully’ increased from 3.9% to 37.0% as the condition deteriorated, with the largest increase occurring when decision-making capacity was lost (10.3% to 23.0%). Predictors of choosing ‘measures to help me die peacefully’ at any stage were: previous personal experience (OR = 1.34, p<0.010), and older age (OR = 1.09 per decade, p<0.010). Negative predictors: living with children (OR = 0.72, p<0.010) and being of “black” race/ethnicity (OR = 0.45, p<0.001).

**Conclusions:**

Public opinion was uniform between GB and USA, but markedly heterogeneous. Despite contemporaneous capacitous consent providing an essential legal safeguard in most jurisdictions, there was a high prevalence of preference for “measures to end my life peacefully” when decision-making capacity was compromised, which increased as dementia progressed. In contrast, a significant number chose preservation of life at all costs, even in end stage dementia. It is challenging to respect the longstanding values of people with dementia concerning either the inviolability of life or personal autonomy, whilst protecting those without decision-making capacity.

## Introduction

Decisions about medical treatment for people approaching the end of life raise clinical and ethical challenges. More people living longer with multiple comorbidities, and advancing medical technologies offering more treatment options, have led to greater complexity in clinical management and decision-making,[1–3] the more so if the person concerned lacks the capacity to participate in decisions about their care. As the end of life approaches, patients and clinicians may need to decide whether to continue life-sustaining treatments and artificial nutrition and hydration (ANH) which could also prolonging suffering. In some jurisdictions it is permissible to take active measures to end the patient’s life: physician-assisted dying (PAD) is now permitted in 6 European countries, Canada and 5 US States (**Box 1**).

**Box 1. Assisted-dying and euthanasia legislation dying worldwide**

**Switzerland *1942*** Assisted dying is not legal, but Article 115 of the Swiss Penal Code permits assisting suicide if the person does so for “unselfish reasons”. Physicians may prescribe lethal drugs, as long as the recipient takes an active role in drug administration. Not restricted to Swiss residents.

**Japan *1995*** There are no acts, laws or statutes on either euthanasia nor assisted dying; and no prohibitions against it. A court case in 1995, following a death at Tokai University, set out four conditions under which euthanasia may occur: unbearable physical pain; close to death; patient consent; and exhaustion of all other pain relief treatments without success.

**Oregon (USA) *1997*** First jurisdiction to legalise assisted dying. The legislation permits the prescription of lethal drugs to adults with a terminal illness, a life expectancy of six months or less, and a sound state of mind.

**Colombia *1997*** In 1997 the Colombian Constitutional Court decriminalised “mercy homicide”, but the conditions under which it could occur were not outlined until 2015.

**Albania *1999 /2002*** In 1999, Albania responded to the European Health Committee, that there were no laws on euthanasia, and thus no legal sanctions against it. Instead physicians are governed by The Code of Ethics and Medical Deontology (2002 rev. 2012) which forbids active euthanasia but allows intervention at the physician’s discretion when patient is unconscious, with no hope to live.

**Netherlands *2002*** Under Dutch law, any action intended to terminate life is in principle a criminal offence. The only exemption from criminal liability is when a patient is experiencing ‘unbearable suffering’ with no prospect of improvement, and the physician fulfils the statutory ‘due care’ criteria. Patients may use advance directives to request euthanasia if they ever suffer from severe dementia. However, this may only be fulfilled if ‘due care’ is taken and the patient is experiencing unbearable suffering with no prospect of improvement.

**Belgium *2002*** Voluntary euthanasia by lethal injection is legal for patients who are mentally competent and have an incurable condition, including mental illness, that causes them constant and unbearable physical or mental suffering.

**Washington (USA) *2008*** Legislation broadly similar to Oregon, legalising assisted dying.

**Luxembourg *2009*** Euthanasia and assisted suicide legalised for mentally competent adults with a severe and incurable terminal condition, causing constant and unbearable physical or psychological suffering without prospects of improvement.

**Montana (USA) *2009*** A physician may raise a defence of ‘consent’ if charged with assisting suicide, Baxter v. Montana 2009.

**Vermont (USA) *2014*** Legislation broadly similar to Oregon, legalising assisted dying.

**California (USA) *2015*** Legislation broadly similar to Oregon, legalising assisted dying.

**South Africa** 2015 Assisting suicide remains illegal. However, in 2015 a Pretoria High Court granted a man with terminal cancer an order that would allow a doctor to assist him in taking his own life without the threat of prosecution.

**Germany *2015*** The German Bundestag approved assisted suicide for altruistic reasons, in legislation broadly similar to Swiss laws but banned the commercialisation of assisted suicide.

**Canada *2016*** Assisting a person with suicide was illegal under section 241(b) of the Canadian Criminal Code. However, in 2015 the Supreme Court of Canada declared section 241(b) invalid and from 2016 physician assisted suicide will be legal for a competent adult, who clearly consents to the termination of life and who has grievous and irremediable condition.

PAD encompasses physician-assisted suicide (PAS) and voluntary euthanasia (VE) (Box 2). Voluntary euthanasia for people with dementia is now permissible in the Netherlands, for those who have made Advance Directives requesting it.[4] **(Box 2)**.

**Box 2. Terminology on assisted dying**

**Assisted dying.** Prescribing life-ending drugs for terminally ill, mentally competent adults to administer themselves after meeting strict legal safeguards. Defined in this way, it is legal and regulated in the US states of Oregon, Vermont, and Washington. It is what the recent Assisted Dying Bill in England and Wales proposed.

**Assisted suicide.** Giving assistance to die to disabled and other people who are not dying, in addition to patients with terminal illness. Drugs are self-administered. Defined in this way, assisted suicide is permitted in Switzerland. Some opponents of the Assisted Dying Bill in England and Wales do not accept “assisted dying” as distinctly different.

**Voluntary euthanasia.** Doctor administration of life-ending drugs to a patient who has given consent. Defined in this way, voluntary euthanasia is permitted in the Netherlands and Belgium.

(Source: White, Dyer and Radar, *BMJ 2*015;351

Debate surrounding PAD goes to the heart of clinical ethical principles: beneficence versus non-maleficence and a patient’s right to autonomy.[5] Critics have argued “physician-assisted suicide is fundamentally inconsistent with the physician's professional role”.[6, 7] Conversely, those who support PAD argue a prolonged death could increase unnecessary physical and psychological suffering, and that patients should have the right to autonomy over their own bodies.[8] Further concerns involve the “slippery slope” and protecting vulnerable persons.[9] The Oregon and Dutch models incorporate consent and disability criteria as safeguards for the vulnerable (**Box 3**).

**Box 3. Common safeguards in the Oregon and Dutch models of Physician Assisted Dying (PAD)**

**Consent criteria:** In most jurisdictions, contemporaneous consent by patients judged to have the requisite mental capacity forms an important safeguard to ensure that no one is unjustly deprived of their right to life. In the Netherlands, an Advance Directive (“living will”) fulfils the consent criterion for patients with dementia, is a notable exception to the requirement for contemporaneous consent.

**Disability criteria:** Physician-assisted dying (PAD) in the USA, and Voluntary Euthanasia (VE) and Physician-assisted Suicide (PAS) in Luxembourg are only legal in cases of established terminal illness. In the Netherlands and Belgium, VE and PAS are permitted in cases of ‘unbearable suffering’ even if the patient is not expected to die within the foreseeable future.

Public policy and legislation are designed to guide decision-makers through this complex landscape. They address potentially competing human rights, such as the right to life and the right to freedom from degrading treatment. In democratic societies, public opinion is also important for shaping law and policy.[10, 11] There is limited good quality evidence on public attitudes concerning issues related to care at the end of life.[12] Despite recent media attention concerning assisted dying in the UK, USA and Canada, open discussion remains taboo.[13–16] Previous surveys have found strong UK public support for PAD, repeatedly around 70%,[17–20] with 63% and 68% of American UK public respectively in 2015.[17, 21]

As part of a programme of research investigating decision-making concerning nutrition and hydration in progressive neurological diseases associated with varying decision-making capacity, we developed survey instrument to investigate public opinion concerning ANH in those circumstances.

### Aims

To investigate the preferences of members of the public regarding care, treatment and outcome, at different stages of illness towards the end of life, as decision-making capacity and swallowing ability deteriorate due to progressive neurological disease. This paper focuses on preferences for measures to sustain or to end life.

## Methods

Most surveys are limited by employing binary yes/no measures. Vignette design is a nuanced way of exploring respondents’ values and judgments concerning sensitive subject areas.[22, 23] A six-stage vignette featured a fictitious person living in a care home whose abilities in both decision-making capacity and swallowing are declining. In the final stage, the person is bed bound, unable to swallow, spends most of their time asleep and has no capacity to make decisions about their care. The stages were developed from: the research literature, including a systematic review of the international literature on ANH;[24] an observational study of a UK hospital feeding issues team;[25] and the expertise of the research team in decision-making capacity, palliative medicine, feeding issues and medical ethics. The instrument was piloted at research group meetings of academics and clinicians, and was adapted for the lay public by Ipsos MORI, the external research organisation who undertook the survey fieldwork. Ipsos MORI are global research company with specialisms in healthcare, and social and political research. Minor adaptations were made by Ipsos MORI USA: it became an online survey and some terminology was changed e.g. “nursing home” substituted for “care home”.

The dependent variable was changes in care preferences, using a four-point scale: (1) sustain life by using any means necessary, including forced tube insertion for ANH and deprivation of liberty; (2) encourage, but not impose, nutrition and hydration by tube or other means; (3) no ANH intervention but continuation of oral nutrition and hydration as far as possible; and (4) provide measures to help die peacefully. Response category 3 broadly reflected the approach of palliative care. Response category 4 used “provide measures” to imply intent to end life, while avoiding the potentially emotionally loaded terms of PAS, PAD and VE: indicating a preference for death to being sustained with nutrition and hydration, it was designed to allow respondents to interpret the meaning broadly, including PAD, PAS and VE.

All questions were multiple-choice and closed. Respondents were asked to indicate what they would want for themselves, regardless of the current law in their jurisdiction. Demographic information was collected, alongside information concerning previous experience of these issues. The questionnaire is attached in **S1 File.**

### Fieldwork and recruitment

GB and USA fieldwork and recruitment was undertaken by Ipsos MORI, using previously-recruited panels paid to complete regular surveys and representative of national populations. In GB, face-to-face interviews with people aged 15 or over used computer-assisted technology during January and February 2015. In the USA, an online survey was completed by people aged 18 or over during July 2015. Respondents were informed that the stages would become progressively worse and that they were free to refuse to answer any question. To the best of our knowledge, no survey participants declined to take part in our component of their regular surveys: normal calculations of response rate are not appropriate to public panel surveys of this nature.

### Informed consent process

Consent to participate in this survey is a three stage process. Stage (1) when participants are initially recruited to the Ipsos MORI panel, the interviewer explains; the surveys, the process, recruitment, anonymity and withdrawal. They then answer any questions the potential respondent has about the survey and the process. As this is happening, the interviewer assesses whether they are happy that the respondent has the decision-making capacity to proceed with the survey. If they are in any doubt, they do not proceed. If they are happy, they give the potential recruit time to consider and then take verbal consent. Once the respondent has verbally consented, they are asked for their full name (which will not be known by the interviewer until this point). This is then recorded on the handheld electronic CAPI (Computer Assisted Personal Interviewing) device to indicate that the respondent has given their informed consent. For recruitment to the online survey, information about the surveys, the process, recruitment, anonymity and withdrawal is read by the potential recruit on the computer screen. They are then given time to consider, and asked to check a box to indicate they have read and understood the information and agree to take part in an online survey. Stage (2) directly before participating in this questionnaire the respondents are read a statement (or they read it themselves on the screen) which describes: the study topic, the objective, funding, who was conducting the study, and how long it would take. The interviewer then checks verbally that the respondent is happy to proceed, or in the online survey the respondent clicks a box to indicate they are happy to proceed. Stage (3) The process of informed consent is ongoing in this survey. Each question has the option to be declined to be answered, if the participant so wishes. The participant also has the option to withdraw entirely from the survey at any point. For the in-person survey, the interviewer also continues to monitor the participant to check they do not look distressed, if they do they interviewer will give the option to stop the survey.

For young adult respondents, aged 15–17 years, parental or guardian consent was taken as well as the respondent’s own consent. For the US online survey, consent was recorded online after the respondent had read the anonymity and withdrawal information and the study statement. No persons under age 18 years took part in the US online survey. Ethical approval for this procedure was obtained from the University of Cambridge Psychology Research Ethics Committee.

### Statistical analysis

Statistical data analysis was undertaken using SPSS version 22 (IBM Corp 2013). Data were weighted for gender, age, education, ethnicity, work status, and regional area to ensure national population representativeness (**Table 1**). Analyses and reported frequencies are based upon the weighted data set.

**Table 1:** Unweighted survey population percentages (N = 2016) and estimated percentage national population data.

	Great Britain		United States	
	% Survey Population *^1^ (N = 991)	% National population *^2^	% Survey Population *^1^ (N = 1025)	% National population *^3^
**Gender**				
Female	55	51	57	52
Male	45	49	43	48
**Age**				
15/18^1^−24	18	15	12	12
25–34	14	17	14	9
35–44	13	16	16	17
45–54	14	17	23	18
55–64	15	14	21	17
65+	27	21	16	18
**Education**				
Degree or higher	27	24	41	29
**Ethnicity (GB) / Race (US)**^**4**^				
“White”	86	88	81	77
All other groups	14	12	19	23

**Notes**

* Percentages may not sum to total due to rounding.

1. Great Britain sample includes adults 15 years and over. USA sample includes adults 18 years and over.

2. National Readership Survey Data 2014.

3. US Census 2013 and American Community Survey 2013.

4. The term ‘ethnicity’ was used in Great Britain and ‘race’ in the United States. Ethnic/racial groups were self-defined by respondents using pre-defined categories.

The main focus of the analysis presented here is on response categories 1 (sustaining life at all costs, including using force or deprivation of liberty) and 4 (measures to help die peacefully). For these responses we present: (a) descriptive analysis over the 6 patient stages; (b) differences in the proportions of participants selecting the categories across the 6 stages (Cochran's Q test); and (c) differences in proportions of participants selecting the response categories between two consecutive stages (exact McNemar tests).

Further analysis investigated outlying response groups: those who selected measures to preserve life at all costs (response 1) in stage 6 (end-stage dementia), which could be considered contrary to a person’s best interests and; those who selected measures to die peacefully (response 4) in stages 1, 6 or at any stage. A logistic regression model was fitted to test the relationship between the likelihood of choosing these response categories and the independent variables of gender, country, ethnicity/race, university education, previous personal experience, living with children and age in years. Odds ratios are reported.

## Results

### Survey population

Responses were obtained from 2016 people, 1025 (50.8%) USA and 991 (49.2%) UK. Unweighted survey data and the estimated representative proportions of UK and USA are shown in **Table 1.** Representative sampling methods were used in both countries, but USA university education was particularly unrepresentative; 41.3% USA sample had a Bachelor’s degree or higher compared to 29.0% in the US population. Nearly one quarter (23.6%, 476) of respondents lived in a household with children; 41.5% (836) reported previous experience in a professional context or with family members or friends.

### Missing data

For each stage, respondents could answer “don’t know” or not answer at all. The missing data for each stage ranged from 1.9% to 2.8%. Combined missing and ‘don’t know’ data are reported.

### Response behaviour and patient vignette

**Fig 1:**
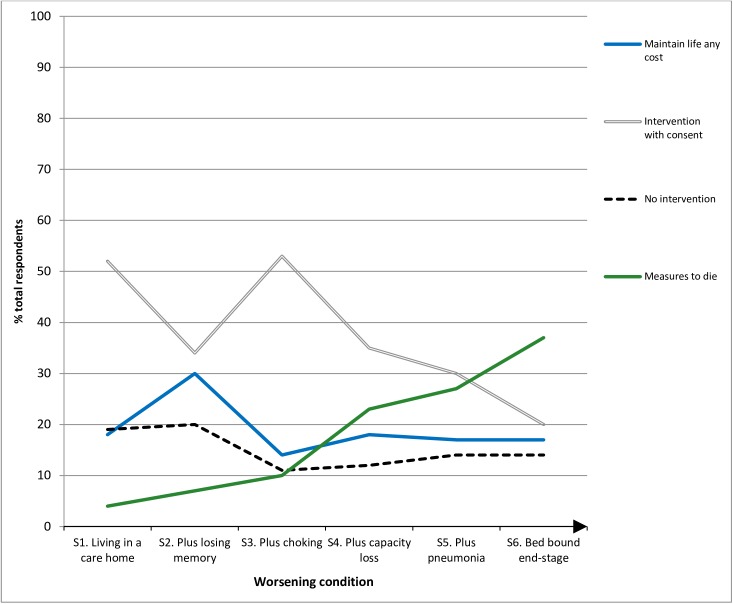
Percentage of respondents’ end of life preferences across six stages of the vignette scenario (N = 2016).

**Table 2** and **Fig 1** summarise responses using weighted data.

**Table 2:** Cross-tabulation illustrating total respondents’ answers across the six-stage vignette using weighted data (n = 2016).

	Scenario 1 *Living in a care home*		Scenario 2 *Losing short-term memory*		Scenario 3 *Choking on food and drink*		Scenario 4 *Very confused & capacity loss*		Scenario 5 *Pneumonia*		Scenario 6 *End stage*, *bed bound*	
	%	n	%	n	%	n	%	n	%	n	%	n
**Response 1** *Sustain life*, *at any cost*	**18**	357	**30**	610	**14**	279	**18**	358	**17**	350	**17**	338
**Response 2** *Intervention w/ consent*	**52**	1045	**34**	690	**53**	1073	**35**	706	**30**	601	**20**	399
**Response 3** *No intervention*	**19**	380	**20**	399	**11**	225	**12**	248	**14**	278	**14**	288
**Response 4** *Measures for ending life*	**4**	78	**7**	133	**10**	208	**23**	463	**27**	536	**37**	745
**Don’t know / Refused**	**8**	157	**10**	184	**13**	279	**12**	236	**13**	251	**13**	246
**Total**	**100**	2016	**100**	2016	**100**	2016	**100**	2016	**100**	2016	**100**	2016

**Notes.**

Weighted data are reported, numbers may not sum to total due to rounding.

### Maintain life at any cost, including the use of force and restraints even when resisted

**Fig 2:**
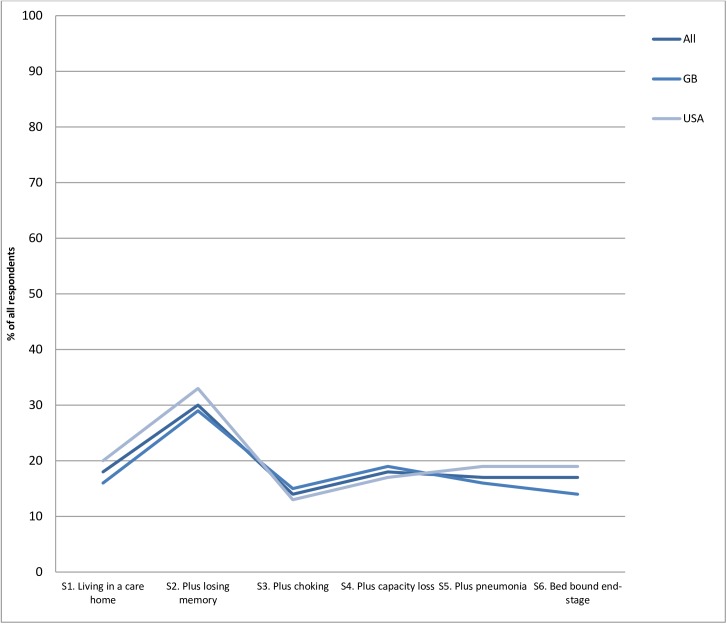
Percentage of respondents choosing maintain life at any cost across six stages (N = 2016).

The proportion of respondents choosing this option peaked at 30.2% at Stage 2 (starting to lose short-term memory), dropped to the lowest point of 13.8% at stage 3 (choking on some food and drink), then remained largely stable over the last three stages (**Fig 2** below).

There was a significant difference in respondents choosing response 1 in all 6 stages (Cochran's Q test, p <0.001), with a significant difference in those choosing response 1 across the first three stages, but not in the last 3 stages (exact McNemar’s tests (p<0.001). The largest increase in those choosing response 1, from 17.7% to 30.2% occurs between stage 1 (living in a care home, to stage 2 (beginning to lose short term memory). The largest decrease, from 30.2% to 13.8%, occurs between stage 2 (beginning to lose short term memory) and stage 3 (choking on food and drink). These changes in proportions choosing response 1 do not differ by country. (**S1 Table**).

### “Measures to help me die peacefully”

**Fig 3:**
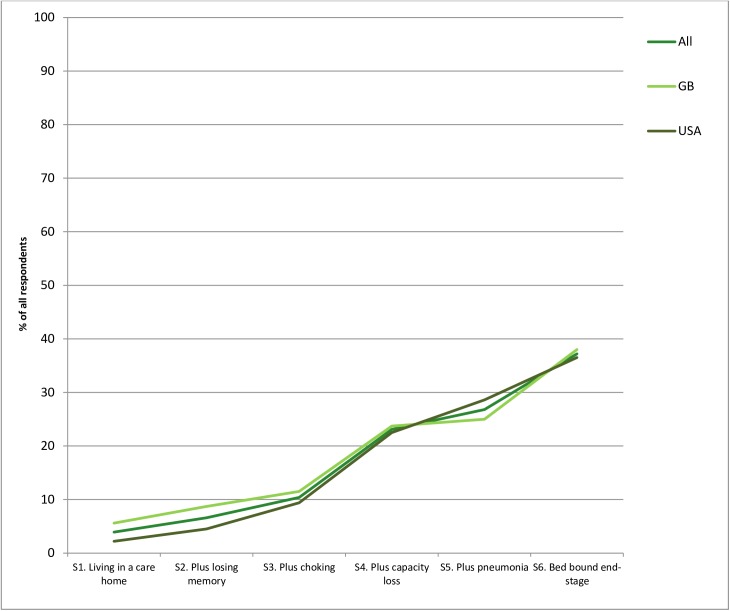
Percentage of respondents choosing “measures to help me die peacefully” across six stages (N = 2016).

The proportions of respondents selecting this option increased significantly between each stage as the person’s condition deteriorated, from 3.9% to 37.0% (Cochran's *Q* test, *p* <0.001) and at each stage (exact McNemar's tests, all *p* <0.001). The largest increase, 10.3% to 23.0%, occurred between stages 3 (some memory loss and choking on food), and 4 (very confused and lost ability to communicate). The second largest increase, 26.6% to 37.0%, occurred between stages 5 (no decision-making capacity and recent aspiration pneumonia) and 6 (end-stage disease, bed bound and asleep). This option was selected on at least one occasion by 44.4% (860) of respondents, and never selected by 55.6% (1079) at any stage (excluding missing cases). (**Fig 3** below, **S2 Table**).

The logistic regression model gave no evidence that gender, country or university education affected whether respondents chose “measures to help me die peacefully” (p>0.1 for all in **S3 Table** and **Table 3** below). Ethnicity was the strongest predictor, with respondents of “black” race/ethnicity much less likely than those of “white” race/ethnicity to choose this response (OR = 0.45). Living in a household with children also reduced the probability of choosing this response (OR = 0.72), while older participants (OR = 1.09 for each 10-year increase in age) and those with previous personal experience (OR = 1.34) were more likely to choose this response. Re-running the analyses including professional experience showed that in contrast to personal experience, professional experience had no statistically association to the response behaviour, in the context of the other predictors. (**S7, S8, S9 and S10 Tables**).

**Table 3:** Logistic regression for “measures to help me die peacefully” chosen at least once (n = 1834).

	Odds Ratio	95% Confidence Interval		*p-value*
		Lower	Upper	
**Country**				0.439
GB	Reference			
US	1.08	0.89	1.31	
**Gender**				0.628
Male	Reference			
Female	0.95	0.79	1.15	
**University education**				0.385
Yes	Reference			
No	1.10	0.89	1.36	
**Ethnicity (GB) / Race (US)**				<0.001
“White”	Reference			
“Black”	0.45	0.30	0.68	
All other groups	0.66	0.48	0.90	
**Experience**				<0.010
No	Reference			
Yes	1.34	1.10	1.62	
**Living with children**				<0.010
No	Reference			
Yes	0.72	0.57	0.90	
**Age**	1.09	1.03	1.15	<0.010
Constant	0.81			0.107

Notes.

Overall model evaluation: Chi square = 68.7, p <0.001.

### Outlying groups analysis

Among the heterogeneous responses obtained, three groups were of particular interest. *Firstly*, 16.8% (337) selected interventions to sustain life at all costs including force and restraints, in end stage disease. Logistic regression identified age, ethnicity and living with children as significant predictors: older participants (OR = 0.78, p<0.001), those living with children (OR = 1.49, p<0.01) and respondents who identified as “black” (OR = 2.57, p<0.001). (**S4 Table**).

*Secondly*, a small proportion of 3.9% (78) chose “measures to help me die peacefully” in the first stage (living in care home, missing meal times for unknown reasons). Logistic regression identified only country as a significant predicator for this response behaviour: USA respondents were significantly less likely than GB respondents to select this option (OR = 0.35, p < .001). (**S5 Table**).

*Thirdly*, 37.0% (745) chose “measures to help me die peacefully” in end-stage disease. Logistic regression identified ethnicity, experience, age and living with children as significant predictors: more likely among older participants (OR = 1.11, p<0.001) and those with past experience (OR = 1.4, p<0.01), and less likely among respondents who did not self-identify as “white” (OR = 0.26, p<0.001) and those living with children (OR = 0.72, p<0.01). (**S6 Table**).

## Discussion

### Heterogeneity of views and the challenge for legislators

This large-scale survey of public opinion found very similar patterns in the views of British and American respondents. One in six (16.8%) expressed a preference for being tube fed when the condition had progressed to near the end of life, a stage where the quality of life available would, to many people, appear minimal. This suggests that a significant minority have a stable moral intuition that life should be supported, even by invasive medical treatment, regardless of the family or medical team’s perception of the individual’s quality of life. This is consistent with a strong, vitalist adherence to the principle of inviolability of human life. On the other hand, over one third (37%) chose “provide measures to help me die peacefully” as their preference for themselves in response to at least one of the stages. This suggests that another, larger, minority hold the view that death would be preferable to being sustained by ANH in these circumstances. This option was kept deliberately broad, to include those opting for a non-interventionist palliative care approach as well as others who would hold that PAD, PAS or VE are morally permissible and desirable under these circumstances.

The challenge for legislators in liberal democracies such as the UK and the US is to enact legal frameworks that represent the views of the majority without unjustly inhibiting the freedom of action of minorities, or putting the lives of people who wish to live at risk.

### Valuing life over liberty

A preference for measures to preserve life at all costs, potentially involving deprivation of liberty peaked in response to stage 2. Around 30% of respondents would prefer to be forced to attend mealtimes, if experiencing short-term memory problems. However, half of these respondents would not wish this coercion to continue if the condition progressed such that being fed by mouth would require greater coercion such as the use of physical restraint. These respondents may be expressing a nuanced moral intuition: generally favouring preservation of life over respecting individual choice, particularly for potentially vulnerable people, provided that this can be achieved without resorting to physical force.

### Valuing liberty over life

The pattern of respondents’ rejection of deprivation of liberty indicates a widespread moral intuition that it is wrong to use force to interfere with the apparently autonomous choices of people with full decision-making capacity. 82% would not want to be made to attend mealtimes if they lived in a care home. However, only around 70% would not want to be forced to attend meals if they developed short-term memory difficulties, suggesting that the moral intuition to respect individual choice is tempered when there is some question about the patient's ability to appreciate the consequences of their choice, even if their capacity to make decisions remained sufficiently intact for the law to require that those decisions be respected.

86% of respondents would not want to be forcibly fed if they could no longer safely swallow ordinary food, higher than at any other stage. This could indicate that, even when there is a question about a person’s ability to consistently appreciate the consequences of not eating, there is a widespread intuition that nutrition should not be imposed if this requires the use of physical force or seems likely to cause distress, even if the potential benefits are considerable. This is consistent with the principle of 'first do no harm' in the Hippocratic oath, or ‘non-maleficence’ in modern medical ethics.[5] The proportion rejecting coercion (force-feeding or tube-feeding) in response to stages 4–6 remains stable at 82% to 83%. This suggests another widespread moral intuition: that when people patients have lost the ability to make decisions for themselves, preservation of life at all costs is not the aim and life-sustaining treatment should be withheld if the risks and burdens are considered to outweigh the benefits.

### Choosing to die

That participants living in households with their children were less likely to choose “measures to help me die peacefully” is consistent with evidence that dependent children reduces suicide risk.[26] Participants self- identifying as belonging to black or minority ethnic groups were also less likely to choose this option, suggesting that moral intuitions regarding care at the end of life, including the permissibility of VE and PAS, are culturally influenced. This introduces additional complexity for legislators in multi-cultural societies, particularly where the ethnic and cultural composition of the legislature or medical profession is different to that of the general population.

Two factors increased the likelihood of respondents expressing a preference for “measures to help me die peacefully”: older age and personal or professional experience of similar illness. This may indicate generational differences in attitudes to end of life care, including VE and PAS, and/or that peoples’ views shift as they witness family and friends ageing and dying. It is of note that in the UK Parliament, the House of Lords (mean age 69 years),[27] voted in favour of legalising AD in January 2015, but the House of Commons (mean age 50 years)[28] voted against in September 2015.

Support for the importance of age as a predictor of attitudes towards PAD comes from a recent cross-sectional survey conducted in the USA in 2015.[29] Periyakoil et al found that older respondents were significantly more supportive of PAD, and that those who reported spiritual or religious beliefs were significantly supportive of PAD. Periyakoil et al also, found that ethnicity and gender were not significant predictors.[29] In contrast, and as discussed above, the findings from this research revealed ethnicity as a strong predictor of choosing ‘measures to help me die peacefully’. Respondents who self-identified as ‘black’ or non-white were less likely to select this option. This difference may be because of the narrower geographical location of respondents in Periyakoil et al (only Hawaii and California) compared with a representative sample of all US states in this research, or it could be because of the different descriptions in each survey–something known to affect outcome in studies on PAD.[30] Periyakoil et al focused solely on PAD, were as the survey instrument in this research examined a broader concept including PAS, PAD and VE.

It appears that most people who would consider death to be preferable to artificially sustained life, including some wishing a form of PAS or VE for themselves, would only prefer this at a late stage of illness, when the quantity and quality of remaining life is limited. The largest increases in preference for “measures to help me die peacefully” occurred at stage 4 (person loses capacity to make decisions about treatment) and stage 6 (condition progresses to terminal phase).

The ‘Oregon model’ of PAD would not be possible for people in either of those situations. As well as the capacity criterion not being met at both stages, in stage 4 the disability criterion (terminal illness) would not be met, while in stage 6, death would be expected to occur naturally within six months, but the person would be unable to self-administer lethal drugs and their only option for hastening death would be euthanasia.

In jurisdictions that permit PAS or VE, their availability depends upon the wording of the disability and capacity criteria. People with incurable cancer commonly retain decision-making capacity until the late stages: they would have the option of PAD under the Oregon model, and PAS or VE under the Dutch model. People with progressive neurological diseases such as multiple sclerosis or motor neurone disease, however, may experience “unbearable suffering” well in advance of the terminal phase. PAS and VE would be available to them in the Netherlands and Belgium, but not the US or Luxembourg. By the terminal phase their physical disabilities would, in all likelihood, prevent them from ending their own life by PAD: VE would be the only option for hastening death, and would be permitted in Luxembourg but not in the US. The risk of permitting PAS but not VE for unbearable suffering is that people would have to choose between using PAS to end their life earlier than they may have wished to or losing altogether the opportunity to hasten their own death in the terminal phase of illness.

People with dementia experience a long and unpredictable illness course: the relatively early loss of decision-making capacity would require either the Swiss model of allowing PAS in early illness, or the Dutch model of Advance Directives. The Swiss approach is not compatible with the wishes expressed by the 37% of study respondents preferring measures to help them die in end-stage dementia, but the Dutch approach is.

From a consequentialist perspective, there is no significant difference in the end-stages of dementia between respecting an Advance Directive to refuse ANH and upholding an Advance Directive to consent to VE. However, both of these approaches are open to the criticism that, by allowing a decision to hasten death made in the past, by someone who does not know what it is like to have dementia, does not extend the reach of self-determination. By privileging that past decision, there is a risk of hastening the death of someone who may still enjoy a reasonable quality of life and wants to continue living. Some argue further, that dementia so profoundly changes personality and sense of self that the person who makes an Advance Directive before the condition progresses is not the same person as the one whose death is hastened when they have lost capacity.[31]

An alternative position, that there is a meaningful distinction to be made between (permissible) Advance Directives to refuse ANH and (impermissible) advance consent to VE, is based upon a moral distinction between acts and omissions. In requiring doctors to respect advance refusals, the law does not compel doctors to act in ways contrary to their professional obligation to help and avoid harming patients. Should the law require doctors to respect advance consent for VE, this would compel a doctor to undertake an act that appears to harm their patient. Arguably, VE may not be harmful, if this is what the patient wants and if it ends, rather than causes, suffering. However this is less clear with VE authorised by Advance Directive, as the person may have changed their mind after developing dementia.

The Oregon model of PAD would permit the provision of help to die for a small number of people, whilst the disability criterion based on terminal illness and the consent criterion based on contemporaneous, capacitous consent limit the potential for harm via the ‘practical slippery-slope’. However, these same safeguards would make help to die unavailable in the situations where, according to our findings, some people may want it. Potentially, this discriminates against people with conditions that limit either their physical ability to self-administer lethal drugs, or their capacity to consent to PAS or euthanasia when they reach the terminal phase of illness.

Advance directives provide a potential solution. Advance decisions to refuse ANH are legally binding, even if ANH is believed to be in the patient’s best interests. The Dutch model could be adapted to protect the choices of other people who want an approach that their physician may disagree with, to be delivered after they have lost decision-making capacity, be that the continuation of ANH or euthanasia.

The limitations of this study are that the survey instrument did not ask people directly about their religious/spiritual views, which other recent research has shown to be an important predictive factor.[29] Another limitation is the lack of exploration of the impact of household income and socioeconomic status of respondents in light of the different healthcare systems in the USA and GB. Further research could investigate these issues in greater depth.

## Conclusions

This survey reveals varied and nuanced views among the US and GB public regarding medical treatment towards the end of life, including ANH, in incurable progressive conditions. Our analysis highlights the difficulties inherent in attempting to incorporate these views into legal regulation of end-of-life care. Current legal frameworks provide stronger protection for those who wish to refuse ANH near the end of life than for those who would request it. Philosophical and practical objections to the use of advance decisions in dementia persist.

## Supporting information

S1 File UK questionnaire. (PDF)

S1 Table Respondents selecting “measures to sustain life at any cost” by country and scenario stage (N = 2016). (PDF)

S2 Table Respondents selecting “measures to help me die peacefully” by country and scenario stage (N = 2016). (PDF)

S3 Table Univariate analysis for selecting ‘measures to help me die peacefully’ (N = 2016). (PDF)

S4 Table Logistic regression for respondents choosing to sustain life at all costs in the final scenario of end stage disease (n = 1854). (PDF)

S5 Table Logistic regression for respondents choosing “measures to help me die peacefully” in the first scenario, living in a care home and missing meals for an unknown reason (n = 1861). (PDF)

S6 Table Logistic regression for respondents choosing “measures to help me die peacefully” in the final scenario of end stage disease (n = 1854). (PDF)

S7 Table Logistic Regression analysis of response behaviour for participants choosing response 4 “measures to help me die peacefully” at least once across the 6 scenarios (n = 1834). (PDF)

S8 Table Logistic Regression of respondents choosing response 4 “measures to help me die peacefully” in scenario 1 (n = 1861). (PDF)

S9 Table Logistic Regression of respondents choosing response 1 “sustain life at any cost” in scenario 6 (n = 1854). (PDF)

S10 Table Logistic Regression of respondents choosing response 4 “measures to help me die peacefully” in scenario 6 (n = 1854). (PDF)
