# Supplementary material for: Preferences for care towards the end of life when decision-making capacity may be impaired: A large scale cross-sectional survey of public attitudes in Great Britain and the United States
Source: PLoS One. 2017 Apr 5;12(4):e0172104. doi: 10.1371/journal.pone.0172104 (PMC5381758; doi:10.1371/journal.pone.0172104)
Supplement: S1 File — (PDF) [file pone.0172104.s001.pdf]

# S1 File: UK questionnaire

## Feeding interventions, decision-making capacity and end of life questionnaire

**PRE-SURVEY TEXT**

**The next questions are part of a study being carried out by the Primary Care Unit at the University of Cambridge for academic purposes. This research is funded by the Dunhill Medical Trust. The aim of the survey is to learn more about the public's views of the care and treatment of people who may have difficulty with eating and drinking due to neurological conditions, and includes questions about care for people nearing the end of life. It will take about 6 minutes to complete this survey.**

**INTRODUCTION**
**ASK ALL**
Over the following set of questions, a scenario will unfold which gets progressively more serious. It concerns a fictitious person living in a care home whose health is declining.

For each question, we would like you think about the person in the scenario described. Then, forgetting about the current law in the UK, choose the option you would most want for yourself, if you were in their situation

Please remember that you do not have to answer any questions if you do not wish to and that you are free to stop answering the questions at any point.

**Q1**
**ASK ALL**
A person has been living in a care home for about a year. They are usually able to care for themselves, and make decisions about their own care. Over the past month however, they have been missing mealtimes. If you were in this situation, what would you want for yourself?

(a) Make me attend mealtimes, even if I resist, so that I have the opportunity to eat
(b) Encourage me to attend mealtimes and eat, but allow me to miss meals if I choose to do so
(c) Serve my meals as usual, and accept if I do not attend mealtimes
(d) Provide measures to help me die peacefully
(e) Don't know
(f) Refused

**Q2**
**ASK ALL**
The person is now beginning to lose their short term memory. As well as missing mealtimes, they sometimes get confused and forget recent events. At times, they can no longer make decisions about their own care. If you were in this situation, what would you want for yourself?

(a) Make me attend mealtimes, even if I resist, so that I have the opportunity to eat

(b) Encourage me to attend mealtimes and eat, but allow me to miss meals if I choose to do so
(c) Serve my meals as usual, and accept if I do not eat them
(d) Provide measures to help me die peacefully
(e) Don't know
(f) Refused

**Q3**
**ASK ALL**
The person is now choking on some food and drink. The medical team have said this could be dangerous, and recommend specially thickened food and drink to help prevent choking. However, the person is refusing the thickened food and drink. They sometimes get confused and forget recent events. At times, they can no longer make decisions about their care. If you were in this situation, what would you want for yourself?

(a) Spoon-feed me the thickened food and drink, using restraints if necessary, so that I eat and drink safely
(b) Help me to eat the thickened food and drink. Provide verbal encouragement, but allow me to make my own choices, even if this means I miss meals and drinks, or choke on the unthickened food and drinks
(c) Serve my meals and drinks as usual, and accept if I do not eat them, or I choke on them
(d) Provide measures to help me die peacefully
(e) Don't know
(f) Refused

**Q4**
**ASK ALL**
The person has now become very confused. They are losing their ability to speak and communicate with the world around them. They cannot make any decision for themselves about their care. The person is now being given specially thickened food and drink to help prevent choking. However the person is often refusing the thickened food. If you were in this situation, what would you want for yourself?

(a) Spoon-feed me the thickened food and drink, using restraints if necessary, so that I eat and drink safely
(b) Help me to eat the thickened food and drink. Provide verbal encouragement, but allow me to make my own choices, even if this means I miss meals and drinks, or choke on the unthickened food and drinks
(c) Serve my meals and drinks as usual, and accept if I do not eat them or I choke on them
(d) Provide measures to help me die peacefully
(e) Don't know
(f) Refused

**Q5**
**ASK ALL**
The person recently had pneumonia caused by choking on the thickened food and drink. The medical team have said it is now dangerous for them to have any food or drink by mouth. They have recommended a one-off procedure to place a feeding tube into the stomach, but this would stop the person eating, and they still seem to enjoy thickened food

on the few occasions that they take it. The person is very confused and cannot make any decision for themselves about their care. If you were in this situation, what would you want for yourself?

(a) Insert the feeding tube, using restraints if necessary, so I can receive food and fluids safely through the tube
(b) Only insert the feeding tube if I seem to accept it, even if this means I would not be able to eat or drink at all
(c) Do not place the feeding tube, but encourage me to eat thickened food and drink, even if I choke
(d) Provide measures to help me die peacefully
(e) Don’t know
(f) Refused

**Q6**
**ASK ALL**
A feeding tube was not inserted. The person is now bed-bound and spends most of their time asleep. They cannot do anything for themselves. The medical team have said it is dangerous for them to have any food or drink by mouth. The medical team have recommended a one-off procedure to place a feeding tube into the stomach, but this would stop the individual eating, and they still seem to enjoy the occasional time they are woken for spoon-feeding. The individual is very confused and cannot make any decision for themselves. If you were in this situation, what would you want for yourself?

(a) Insert the feeding tube, using restraints if necessary, so I can receive food a fluids safely through the tube, even if this means I spend most of my time asleep
(b) Only insert the feeding tube if I seem to accept it, even if this means I would not be able to eat and drink at all, and this means I spend most of my time asleep
(c) Do not place the feeding tube, but continue to wake me for spoon-feeding of food or fluids, even if I choke
(d) Provide measures to help me die peacefully
(e) Don’t know
(f) Refused

**Q7**
**ASK ALL**
In the last of the six scenarios, the person’s difficulty with swallowing and making decisions had become very advanced. They spent most of their time asleep and were unable to make decisions for themselves, or communicate with the world around them. In a situation like this, what do you think are the most important considerations? Please choose the three factors you think are most important.

(a) I want to be free from pain and discomfort
(b) I want to live for as long as possible into old age
(c) I want my dignity to be respected
(d) I want to avoid a long drawn-out death
(e) I want measures to help me die peacefully
(f) Don’t know
(g) Refused

**Q8**
**ASK ALL**
In the last of the six scenarios, the person spent most of their time asleep and was unable to make decisions for themselves, or communicate with the world around them. However, when they were able to, they expressed their wishes about what they wanted to happen at the end of their life. Their family, friends and the medical team also had a range of different opinions about what should be done. Whose views are the most important in a situation like this? Please choose the three factors you think are most important.

a) The patient’s previously expressed wishes
b) The wishes of the patient’s partner/husband/wife
c) The wishes of the patient’s children
d) The wishes of other family members
e) The wishes of close friends and loved ones
f) The medical team’s recommendations
g) The views of religious or spiritual advisors
h) Don’t know
i) Refused

**Q9**
**ASK ALL**
Have you had previous experience of these issues?

a) Yes, with a family member or person close to me
b) Yes, in a professional context
c) No
d) Unsure
e) Refused

**Q10**
**ASK ALL**
What else would be important to you if you were ever in any of these situations?

**OPEN ENDED**

(a) Don’t know
(b) Refused
