# Supplementary material for: Preferences for care towards the end of life when decision-making capacity may be impaired: A large scale cross-sectional survey of public attitudes in Great Britain and the United States
Source: PLoS One. 2017 Apr 5;12(4):e0172104. doi: 10.1371/journal.pone.0172104 (PMC5381758; doi:10.1371/journal.pone.0172104)
Supplement: S1 Table — (PDF) [file pone.0172104.s002.pdf]

S1 Table: Respondents selecting "measures to sustain life at any cost" by country and scenario stage (N=2016)

**Response 1:** *Measures to sustain life at any cost*	**Scenario 1:** ***Living in a care home***		**Scenario 2:** ***Losing short-term memory***		**Scenario 3:** ***Choking on food and drink***		**Scenario 4:** ***V. confused & capacity loss***		**Scenario 5:** ***Pneumonia***		**Scenario 6:** ***End stage, bed bound***	
	**Within country %**	n	**Within country %**	n	**Within country %**	n	**Within country %**	n	**Within country %**	n	**Within country %**	n
**All**	**18**	357	**30**	610	**14**	279	**18**	358	**17**	350	**17**	338
**GB**	**16**	153	**29**	273	**15**	144	**19**	187	**16**	159	**14**	138
**USA**	**20**	204	**33**	337	**13**	135	**17**	171	**19**	191	**19**	199

Notes

Weighted data are reported, numbers may not sum to total due to rounding
