# Supplementary material for: Preferences for care towards the end of life when decision-making capacity may be impaired: A large scale cross-sectional survey of public attitudes in Great Britain and the United States
Source: PLoS One. 2017 Apr 5;12(4):e0172104. doi: 10.1371/journal.pone.0172104 (PMC5381758; doi:10.1371/journal.pone.0172104)
Supplement: S2 Table — (PDF) [file pone.0172104.s003.pdf]

S2 Table: Respondents selecting "measures to help me die peacefully" by country and scenario stage (N=2016)

**Response 1:** *Provide measures to help me die peacefully*	**Scenario 1:** ***Living in a care home***		**Scenario 2:** ***Losing short-term memory***		**Scenario 3:** ***Choking on food and drink***		**Scenario 4:** ***V. confused & capacity loss***		**Scenario 5:** ***Pneumonia***		**Scenario 6:** ***End stage, bed bound***	
	**Within country %**	n	**Within country %**	n	**Within country %**	n	**Within country %**	n	**Within country %**	n	**Within country %**	n
**All**	**4**	78	**7**	133	**10**	208	**23**	463	**27**	536	**37**	745
**GB**	**6**	55	**9**	87	**12**	112	**24**	232	**26**	243	**39**	371
**USA**	**2**	23	**5**	46	**10**	96	**23**	231	**29**	293	**37**	374

Notes

Weighted data are reported, numbers may not sum to total due to rounding
