# Supplementary material for: Preferences for care towards the end of life when decision-making capacity may be impaired: A large scale cross-sectional survey of public attitudes in Great Britain and the United States
Source: PLoS One. 2017 Apr 5;12(4):e0172104. doi: 10.1371/journal.pone.0172104 (PMC5381758; doi:10.1371/journal.pone.0172104)
Supplement: S3 Table — (PDF) [file pone.0172104.s004.pdf]

S3 Table: Univariate analysis for selecting 'measures to help me die peacefully' (N=2016)

Response 4 *Help me die peacefully* chosen at least once	Total sample		Country		Gender		Ethnicity (GB) / Race (US)		Education		Experience with family or friend		Living with children		Age	
	(N)	GB (n)	US (n)	Male (n)	Female (n)	'White' (n)	'Black' (n)	Other (n)	Uni degree (n)	No degree (n)	Yes (n)	No (n)	Yes (n)	No (n)	M	SD
**No**	1079	528	551	517	562	825	99	155	290	752	67	689	306	773	44.21	17.35
**Yes**	860	423	437	415	445	745	39	76	211	614	369	482	169	692	48.33	17.94
**Total**	1939	951	988	932	1007	1570	138	231	501	1366	736	1171	475	1465	n/a	n/a

Notes
Weighted data are reported, numbers may not sum to total due to rounding
