# Supplementary material for: Preferences for care towards the end of life when decision-making capacity may be impaired: A large scale cross-sectional survey of public attitudes in Great Britain and the United States
Source: PLoS One. 2017 Apr 5;12(4):e0172104. doi: 10.1371/journal.pone.0172104 (PMC5381758; doi:10.1371/journal.pone.0172104)
Supplement: S4 Table — (PDF) [file pone.0172104.s005.pdf]

S4 Table: Logistic regression for respondents choosing to sustain life at all costs in the final scenario of end stage disease (n=1854)

	Odds Ratio	95% Confidence Interval		*p-value*
		Lower	Upper	
**Country**				0.910
GB	Reference			
US	1.25	0.97	1.61	
**Gender**				0.330
Male	Reference			
Female	1.13	0.88	1.45	
**University education**				
Yes	Reference			0.769
No	0.96	0.73	1.27	
**Ethnicity (GB) / Race**				<0.001
"White"	Reference			
"Black"	2.57	1.73	3.82	
All other groups	1.30	0.91	1.84	
**Experience**				0.662
No	Reference			
Yes	1.06	0.82	1.37	
**Living with children**				<0.01
No	Reference			
Yes	1.49	1.14	1.96	
**Age**	0.78	0.72	0.85	<0.001
Constant	0.05			

Note: Overall model evaluation: Chi square = 99.498 , p <0.001
