# Supplementary material for: Preferences for care towards the end of life when decision-making capacity may be impaired: A large scale cross-sectional survey of public attitudes in Great Britain and the United States
Source: PLoS One. 2017 Apr 5;12(4):e0172104. doi: 10.1371/journal.pone.0172104 (PMC5381758; doi:10.1371/journal.pone.0172104)
Supplement: S5 Table — (PDF) [file pone.0172104.s006.pdf]

S5 Table: Logistic regression for respondents choosing "measures to help me die peacefully" in the first scenario, living in a care home and missing meals for an unknown reason (n=1861)

	Odds Ratio	95% Confidence Interval		*p-value*
		Lower	Upper	
**Country**				<0.001
GB	Reference			
US	0.35	0.21	0.60	
**Gender**				0.615
Male	Reference			
Female	0.89	0.55	1.43	
**University education**				0.752
Yes	Reference			
No	0.75	0.45	1.26	
**Ethnicity (GB) / Race**				0.401
"White"	Reference			
"Black"	0.49	0.11	2.30	
All other groups	0.58	0.21	1.59	
**Experience**				0.704
No	Reference			
Yes	1.10	0.68	1.79	
**Living with children**				0.286
No	Reference			
Yes	0.70	0.36	1.35	
**Age**	1.01	0.97	1.29	0.128
Constant	0.05			

Note: Overall model evaluation: Chi square = 68.7 , p <0.001
