# Supplementary material for: Preferences for care towards the end of life when decision-making capacity may be impaired: A large scale cross-sectional survey of public attitudes in Great Britain and the United States
Source: PLoS One. 2017 Apr 5;12(4):e0172104. doi: 10.1371/journal.pone.0172104 (PMC5381758; doi:10.1371/journal.pone.0172104)
Supplement: S6 Table — (PDF) [file pone.0172104.s007.pdf]

S6 Table: Logistic regression for respondents choosing "measures to help me die peacefully" in the final scenario of end stage disease (n=1854)

	Odds Ratio	95% Confidence Interval		*p-value*
		Lower	Upper	
**Country**				0.635
GB	Reference			
US	1.05	0.86	1.28	
**Gender**				0.832
Male	Reference			
Female	0.98	0.81	1.19	
**University education**				0.652
Yes	Reference			
No	1.05	0.85	1.31	
**Ethnicity (GB) / Race**				<0.001
"White"	Reference			
"Black"	0.26	0.16	0.43	
All other groups	0.61	0.44	0.84	
**Experience**				<0.01
No	Reference			
Yes	1.40	1.15	1.71	
**Living with children**				<0.01
No	Reference			
Yes	0.72	0.57	0.92	
**Age**	1.11	1.05	1.18	<0.001
Constant	0.39			

Note: Overall model evaluation: Chi square =103.52 , p <0.001
