# Supplementary material for: Preferences for care towards the end of life when decision-making capacity may be impaired: A large scale cross-sectional survey of public attitudes in Great Britain and the United States
Source: PLoS One. 2017 Apr 5;12(4):e0172104. doi: 10.1371/journal.pone.0172104 (PMC5381758; doi:10.1371/journal.pone.0172104)
Supplement: S7 Table — (PDF) [file pone.0172104.s008.pdf]

S7 Table: Logistic Regression analysis of response behaviour for participants choosing response 4 "measures to help me die peacefully" at least once across the 6 scenarios (n=1834)

		B	S.E.	Wald	df	Sig.	Exp(B)	95% C.I.for EXP(B)	
								Lower	Upper
Step 1[a]	age_centered	.084	.029	8.219	1	.004	1.087	1.027	1.152
	Country(1)	.077	.098	.611	1	.434	1.080	.891	1.309
	Gender(1)	-.039	.097	.167	1	.683	.961	.795	1.162
	Uni_education(1)	.082	.110	.558	1	.455	1.085	.876	1.345
	Ethnicity			19.731	2	.000			
	Ethnicity(1)	-.797	.207	14.871	1	.000	.451	.301	.676
	Ethnicity(2)	-.423	.158	7.124	1	.008	.655	.481	.894
	Exp_fam(1)	.287	.099	8.403	1	.004	1.332	1.097	1.617
	EXP_PROF(1)	-.235	.206	1.293	1	.255	.791	.528	1.185
	Child_household(1)	-.323	.118	7.477	1	.006	.724	.574	.912
	Constant	-.187	.129	2.101	1	.147	.829		

a. Variable(s) entered on step 1: age_centered, Country, Gender, Uni_education, Ethnicity, Exp_fam, EXP_PROF, Child_household.
