# Supplementary material for: Preferences for care towards the end of life when decision-making capacity may be impaired: A large scale cross-sectional survey of public attitudes in Great Britain and the United States
Source: PLoS One. 2017 Apr 5;12(4):e0172104. doi: 10.1371/journal.pone.0172104 (PMC5381758; doi:10.1371/journal.pone.0172104)
Supplement: S8 Table — (PDF) [file pone.0172104.s009.pdf]

S8 Table: Logistic Regression of respondents choosing response 4 “measures to help me die peacefully” in scenario 1 (n=1861)

		B	S.E.	Wald	df	Sig.	Exp(B)	95% C.I.for EXP(B)	
								Lower	Upper
Step 1[a]	age_centered	.111	.073	2.320	1	.128	1.117	.969	1.289
	Country(1)	-1.045	.271	14.884	1	.000	.352	.207	.598
	Gender(1)	-.137	.245	.311	1	.577	.872	.540	1.410
	Uni_education(1)	-.267	.265	1.019	1	.313	.766	.456	1.286
	Ethnicity			1.793	2	.408			
	Ethnicity(1)	-.704	.787	.799	1	.372	.495	.106	2.315
	Ethnicity(2)	-.545	.517	1.108	1	.292	.580	.210	1.599
	Exp_fam(1)	.102	.249	.167	1	.683	1.107	.680	1.803
	EXP_PROF(1)	.296	.466	.404	1	.525	1.345	.540	3.352
	Child_household(1)	-.375	.339	1.226	1	.268	.687	.353	1.335
	Constant	-2.412	.294	67.474	1	.000	.090		

a. Variable(s) entered on step 1: age_centered, Country, Gender, Uni_education, Ethnicity, Exp_fam, EXP_PROF, Child_household.
