# Supplementary material for: Preferences for care towards the end of life when decision-making capacity may be impaired: A large scale cross-sectional survey of public attitudes in Great Britain and the United States
Source: PLoS One. 2017 Apr 5;12(4):e0172104. doi: 10.1371/journal.pone.0172104 (PMC5381758; doi:10.1371/journal.pone.0172104)
Supplement: S9 Table — (PDF) [file pone.0172104.s010.pdf]

S9 Table: Logistic Regression of respondents choosing response 1 “sustain life at any cost” in scenario 6 (n=1854)

		B	S.E.	Wald	df	Sig.	Exp(B)	95% C.I.for EXP(B)	
								Lower	Upper
Step 1[a]	age_centered	-.247	.040	37.284	1	.000	.781	.722	.846
	Country(1)	.221	.131	2.830	1	.093	1.247	.964	1.612
	Gender(1)	.121	.127	.909	1	.340	1.128	.880	1.446
	Uni_education(1)	-.035	.142	.061	1	.805	.965	.730	1.276
	Ethnicity			22.069	2	.000			
	Ethnicity(1)	.946	.203	21.719	1	.000	2.575	1.730	3.833
	Ethnicity(2)	.264	.179	2.183	1	.140	1.302	.917	1.848
	Exp_fam(1)	.058	.131	.193	1	.661	1.059	.819	1.370
	EXP_PROF	.098	.255	.148	1	.700	1.103	.669	1.820
	Child_household(1)	.398	.138	8.375	1	.004	1.489	1.137	1.949
	Constant	-2.140	.177	145.639	1	.000	.118		

a. Variable(s) entered on step 1: age_centered, Country, Gender, Uni_education, Ethnicity, Exp_fam, EXP_PROF, Child_household.
